# The Interacting Head Motif Structure Does Not Explain the X-Ray Diffraction Patterns in Relaxed Vertebrate (Bony Fish) Skeletal Muscle and Insect (*Lethocerus*) Flight Muscle

**DOI:** 10.3390/biology8030067

**Published:** 2019-09-14

**Authors:** Carlo Knupp, Edward Morris, John M. Squire

**Affiliations:** 1 School of Optometry and Vision Science, Cardiff University, Cardiff CF10 3NB, UK; knuppc@cardiff.ac.uk; 2 Division of Structural Biology, Institute of Cancer Research, London SW7 3RP, UK; Ed.Morris@icr.ac.uk; 3 Muscle Contraction Group, School of Physiology, Pharmacology and Neuroscience, University of Bristol, Bristol BS8 1TD, UK; 4 Computational and Systems Medicine, Faculty of Medicine, Imperial College, London SW7 2BZ, UK

**Keywords:** bony fish muscle, insect flight muscle, myosin filament structure, myosin crossbridge cycle, thick filament activation, interacting head motif

## Abstract

Unlike electron microscopy, which can achieve very high resolution but to date can only be used to study static structures, time-resolved X-ray diffraction from contracting muscles can, in principle, be used to follow the molecular movements involved in force generation on a millisecond timescale, albeit at moderate resolution. However, previous X-ray diffraction studies of resting muscles have come up with structures for the head arrangements in resting myosin filaments that are different from the apparently ubiquitous interacting head motif (IHM) structures found by single particle analysis of electron micrographs of isolated myosin filaments from a variety of muscle types. This head organization is supposed to represent the super-relaxed state of the myosin filaments where adenosine triphosphate (ATP) usage is minimized. Here we have tested whether the interacting head motif structures will satisfactorily explain the observed low-angle X-ray diffraction patterns from resting vertebrate (bony fish) and invertebrate (insect flight) muscles. We find that the interacting head motif does not, in fact, explain what is observed. Previous X-ray models fit the observations much better. We conclude that the X-ray diffraction evidence has been well interpreted in the past and that there is more than one ordered myosin head state in resting muscle. There is, therefore, no reason to question some of the previous X-ray diffraction results on myosin filaments; time-resolved X-ray diffraction should be a reliable way to follow crossbridge action in active muscle and may be one of the few ways to visualise the molecular changes in myosin heads on a millisecond timescale as force is actually produced.

## 1. Introduction

A long-term goal of those working to describe in detail the nature of the crossbridge interaction between myosin and actin filaments that occurs during muscle contraction is to describe the underlying molecular movements using analysis of time-resolved X-ray diffraction patterns [1,2,3]. This kind of analysis is made more practicable by using particular types of muscle, namely simple lattice muscles (e.g., bony fish muscle) for vertebrates [4,5,6] and insect flight muscle for invertebrates [7,8,9]. These muscles are attractive in that the myosin filament organisation within the muscle A-bands can be sufficiently well-ordered that their observed X-ray diffraction patterns are quasi-crystalline and are therefore amenable to the application of standard crystallographic methods of analysis [6,10,11]. Thus, models for crossbridge configurations on the myosin filaments in resting bony fish muscle (Hudson et al. [10]) and resting insect flight muscle (AL-Khayat et al. [11]) have been developed by rigorous fitting of the observed resting X-ray diffraction patterns and modelling of the patterns using known information about filament and lattice symmetry and the shape of the myosin heads. Both of these models have an attractive feature in that the actin-binding domains of some of the myosin heads are relatively close to the sites on actin filaments where they would need to bind. In addition, the heads are oriented with their actin binding sites suitably aligned to interact with the actin filaments with the head orientation observed in 3D reconstructions from electron micrographs of actin filaments labelled with myosin heads in the rigor state (no ATP [12,13]).

X-ray diffraction analysis of this kind requires the setting up of structural models in silico and then altering the parameters describing the models in some form of parameter search until the computed diffraction pattern matches the observed pattern as closely as possible. The adjustable parameters may include the tilt, rotation, slew, and radius of the myosin heads and alterations in their shape around points of flexibility. The goodness of fit is usually described by an R-factor which compares the computed and observed diffraction intensities and scores the result. The model with the lowest R-factor is taken as the best. The R-factor size can be thought of as being described in a multidimensional “mountain range” and there is a potential problem with these searches in that the searching can get stuck in a low valley which looks correct when the actual lowest valley (lowest R-factor) is somewhere else. A local minimum has been found, but the global minimum has been missed. We do not know a priori if this is the case and can only make judgements based on the size of the R-factor compared to the number of free parameters involved in the search, the number of iterations in the parameter search, the apparent plausibility of the chosen structure, and the reproducibility of the structure with the lowest R-factor in independent repetitive searches.

**Figure 1:**
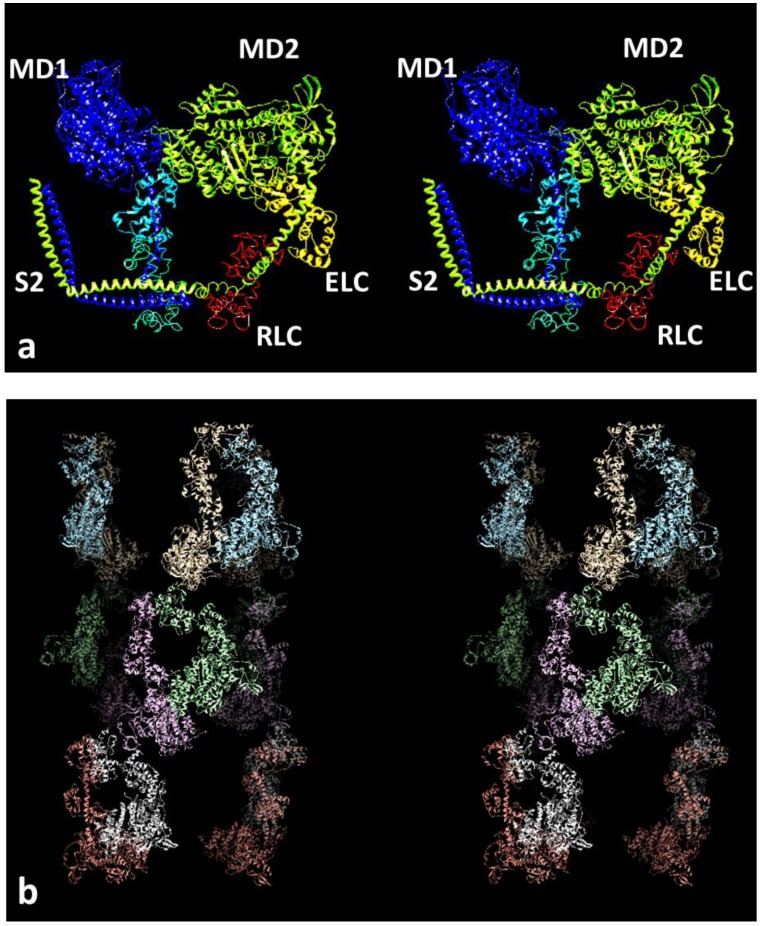
(**a**) The interacting head motif (IHM) of the two myosin heads in a myosin molecule (pdb accession code 3DTP) shown as a stereo pair (wall-eyed stereo). One head in each pair is in shades of blue and the other in yellow. The motor domains are towards the top, and the lever arms with a long central α–helix (each with two light chains, yellow and red on the right head) are below the motor domains. The coiled-coil of part of the subfragment 2 (S2) of the myosin rod, where the two heads join together, is shown at the lower left. The actin binding site on the right head abuts the left head. The right hand head is called the blocked head and the other the free head. (**b**) Stereo image of the reconstruction from electron microscopy and single particle analysis of the myosin filaments in human heart muscle, with heads fitted to the density. The total length of filament shown here is around 43 nm. There are three crowns of heads with six heads in each crown. The head pair configuration is similar to that in (**a**), but the whole structure, for example of the heads nearest to the viewer, has been rotated 180° top to bottom roughly around a horizontal rotation axis in the plane of the page. Other head pairs are then rotated around the filament axis by varying amounts.

Studies of isolated myosin filaments by electron microscopy (EM) have been carried out for many years and 3D reconstructions and other kinds of analysis have revealed the rotational symmetry of these myosin filaments: 3-fold for vertebrates [14,15,16], 4-fold for insect flight muscle [17]. EM and X-ray methods have also shown their axial repeats; a common crown spacing of around 143 to 145 Å and axial repeats of around 430 Å for vertebrate thick filaments [6,7,18] and 1160 Å for insect thick filaments [7]. Other myosin filament symmetries are described elsewhere (e.g., [19,20]). For many years the EM reconstructions were inconclusive because the available resolution was not good enough to enable different head organisations to be properly distinguished. More recently, with improvements in EM techniques, higher resolution studies have homed in on one particular kind of structure for the myosin head arrays in relaxed muscles. This configuration is the so-called interacting head motif (IHM; Figure 1a) first observed in 2D crystals of isolated myosin heads from vertebrate smooth muscle [21]. It was then seen on the thick filaments of tarantula muscle [22] and subsequently has been seen on all myosin filaments that have been studied in enough detail [23], including vertebrate muscle myosin filaments [24,25,26] and insect flight muscle myosin filaments [27]. The important point here is that these interacting head motif structures are different from the structures modelled for the same filaments using X-ray diffraction analysis.

The question we ask here is, therefore, whether the X-ray diffraction modelling was incorrect because the searching had become stuck in a local R-factor minimum and we had missed the interacting head motif structure in our analysis, or whether both structures are in fact sensible but perhaps occur in the same muscle type at different times, under different conditions. In other words, can we rely on the X-ray analysis? We need to know the answer to this before we can attempt to model the whole crossbridge cycle using X-ray diffraction methods. Here we deal with the myosin filament structures in vertebrate striated (i.e., fish skeletal and human cardiac) muscles and insect flight (*Lethocerus*) muscles. We show that the interacting head motif structures explain the observed X-ray diffraction patterns from these resting muscles less well than the original models [10,11] and we discuss the implications of this finding.

## 2. Methods and Results

### 2.1. Strategy

In order to test whether the interacting head motif structures could explain the observed low-angle X-ray diffraction patterns from resting bony fish and insect flight muscles, we generated the full A-band unit cells of just the myosin filaments in fish muscle and insect flight muscle using the observed EM-fitted interacting head motif head configurations arranged with the appropriate myosin filament symmetry from the EM reconstructions of human heart muscle myosin filaments [26] and *Lethocerus* myosin filaments [27]. We used a locally produced program to calculate the expected intensities of all the reflections (Miller indices *h*, *k*, *l*) from the two different unit cells out to a resolution of about 60 Å. Previously we had used the CCP13 program FibreFix [28] to determine the observed intensities and MOVIE [10] to generate the myosin filament models and calculate their diffraction patterns. The new calculations using our home-generated program are totally independent of the previous MOVIE calculations of the diffraction patterns. Having calculated the diffraction patterns, the results from our programs were checked by using Fourier synthesis on the calculated reflection data to confirm that the original myosin filament structures were regenerated.

### 2.2. Bony Fish (Plaice) Muscle Myosin Filaments and Unit Cell

**Figure 2:**
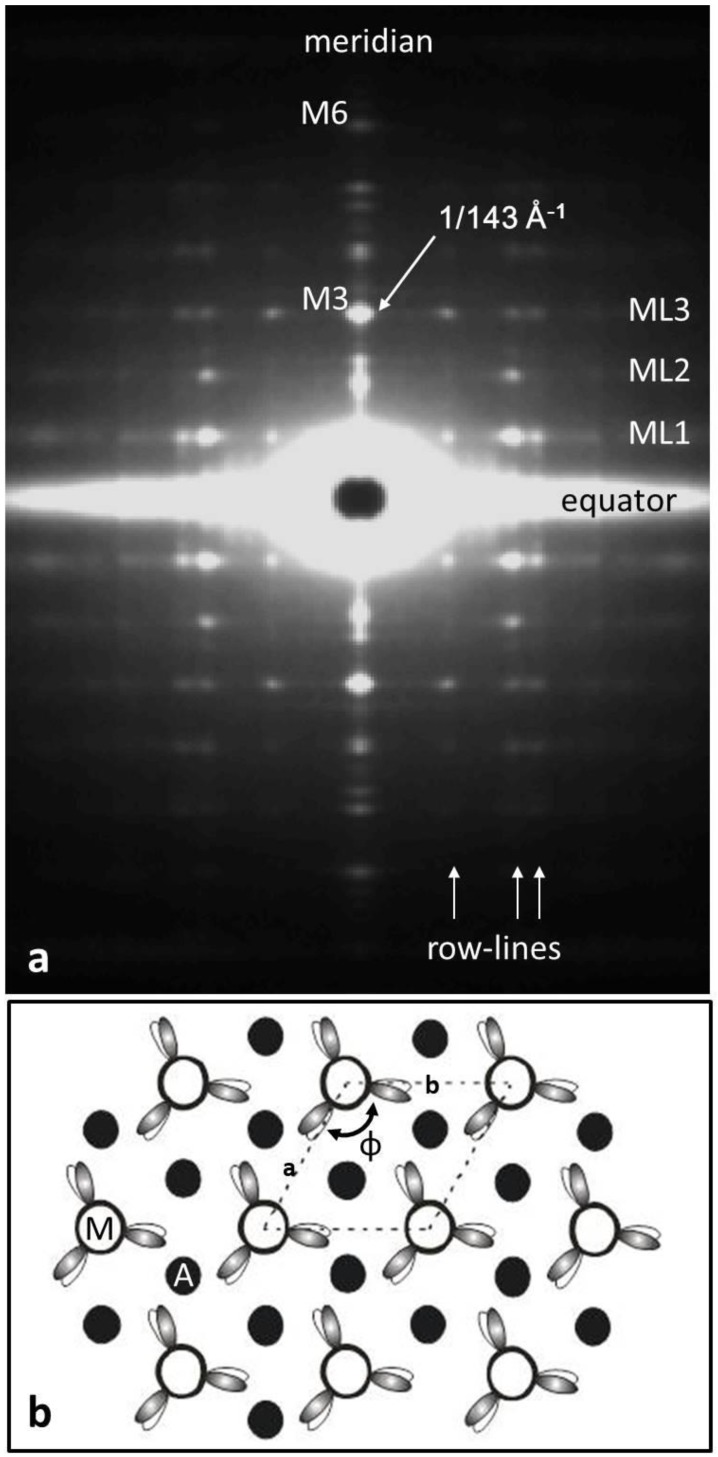
(**a**) The low-angle X-ray diffraction pattern from bony fish muscle (fibre axis vertical) showing the horizontal layer-lines and vertical row-lines on which the observed peaks are situated. Almost all of what can be seen here comes from the myosin filaments in the muscle. (**b**) The “simple lattice” unit cell of the A-band lattice in bony fish muscle viewed down the fibre axis showing the single rotation (N) of the myosin filaments around their long axes and the 3-fold rotational symmetry of the head arrangement on one crown of heads. Actin filament positions (black circles) at the trigonal points are labelled A.

From analysis of the fish muscle X-ray diffraction pattern (Figure 2a) using FibreFix [28] or its predecessor, the resting bony fish (plaice) muscle unit cell (Figure 2b) was found to have dimensions a = b = 470 Å, c = 429.6 Å, α = β = 90°, γ = 120°. It is a simple lattice structure [4,5,6], with every myosin filament having the same rotation around its long axis on every lattice point of the hexagonal A-band unit cell. Actin filaments, with roughly 13/6 helical symmetry, lie at the trigonal points of this lattice (i.e., at the centre of a triangle formed by three myosin filaments). Their axial repeat is around 360 Å, so the actin layer-lines do not directly overlap the myosin layer-lines (e.g., see Figure 2.8 in [29]). In any case, unlike the myosin layer-lines, the actin layer-lines are not sampled; to get accurate myosin layer-line intensities, the actin diffraction pattern can be stripped as part of the continuous background.

### 2.3. The Human Heart Muscle Myosin Filament

A 3D reconstruction of the myosin filaments from human heart was generated by AL-Khayat et al. [26] as a density map to about 25-Å resolution. This was then fitted with the interacting head motif structure [21], which was found to be an almost perfect match to the density. This structure is shown in stereo in Figure 1b. Vertebrate muscle myosin filament symmetry has the head pairs approximately on a 3-start, 9-residue per turn helix of pitch 3 × 429.6 Å [14]. Each residue is a pair of myosin heads. Because of the three equivalent strands and the 3-fold rotational symmetry of this structure, it has a true repeat of 429.6 Å.

There are three “crowns” of myosin heads in this repeat, with the head pairs of three myosin molecules in each crown, but, unlike an ideal helical structure, the three crowns in a 429.6-Å repeat are not quite the same; there is a periodic perturbation [6,18]. This can be seen in Figure 1b, where the axial separation of the head pairs along a long-pitched strand, which would be at regular axial steps of 143.2 Å if the structure was perfectly helical, actually show significant perturbations from this.

### 2.4. Calculation of the X-ray Diffraction Pattern from Vertebrate Muscle

**Figure 3:**
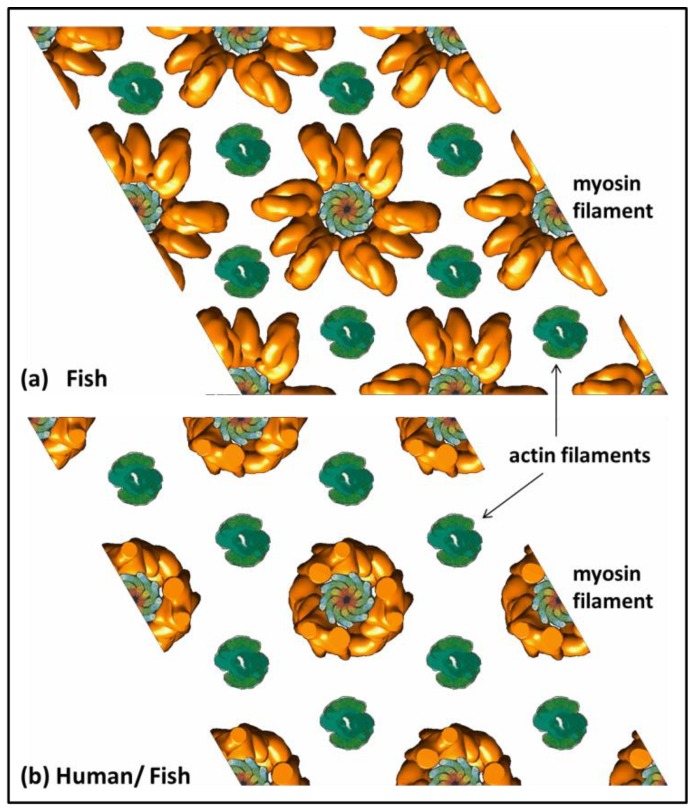
The fish muscle unit cell with the myosin filament head arrangements (dark yellow) from (**a**) Hudson et al. [10] and (**b**) as in human heart muscle (AL-Khayat et al. [25]). The myosin filament backbone structure is from Chew and Squire [30]. Actin filaments are shown in green.

The important myosin filament layer-lines in vertebrate muscle X-ray diffraction patterns (Figure 2a) out to around 60 Å are (*l* =) 1 (429.6 Å), 2, 3 (143.2 Å; M3 meridional and layer line), 4, 5, and 6 (71.6 Å; M6 meridional and layer line). In order to compare the human heart myosin filament structure with the plaice fin muscle diffraction pattern, the human heart myosin filament structure (Figure 1b) was positioned at the unit cell corners of the fish muscle unit cell (Figure 3b) and its diffraction pattern was computed from the formula:

F(*h*,*k*,*l*) = ΣΔ(x,y,z)exp (2πi(*h*x + *k*y + *l*z)) (1)

where *h*, *k*, and *l* are the Miller indices of a particular reflection in the X-ray diffraction pattern and Δ(x,y,z) is the myosin filament density at fractional unit cell coordinates x, y, and z.

Technical note: The observed X-ray diffraction pattern is a rotation pattern from unit cells in sarcomeres at all rotations around the fibre axis. Each spot in the X-ray diffraction pattern is then a sum of everything that occurs at the same radius (R) on a particular layer-line *l*, but with different rotations around the fibre axis—they are multiplets. Even peaks with different Miller indices can occur at the same radius on a layer-line (e.g., the 53 *l* and 70 *l* peaks).]

An uncertainty of this calculation is the absolute rotation of the myosin filament within the unit cell, relative to the unit cell edges. The human thick filament density map or pdb structure was therefore rotated systematically around the c-axis in the fish muscle unit cell in steps of 1° over the range 0 to 60° and for each rotation an R-factor was calculated using the formula:

R = Σ(I_obs_ − I_calc_)^2^/Σ(I_obs_)^2^] (2)

where I_obs_ is the observed intensity and I_calc_ is the intensity calculated from the model structure.

**Figure 4:**
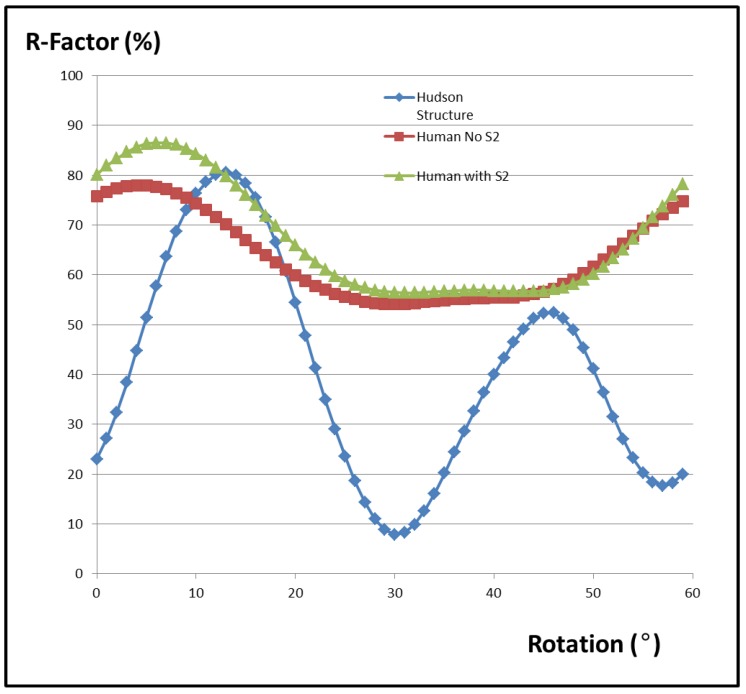
The calculated R-factor from the human heart muscle myosin filament in the fish muscle unit cell (red trace) showing a large variation with rotation angle relative to the unit cell axis. The analogous R-factor for the fish thick filament structure determined by Hudson et al. [10] is in blue. As expected, both R-factors are periodic because of the symmetry in the lattice and they repeat after 60°. Also included is the R-factor of the hybrid human system if the myosin S2 is also included. Inclusion of S2 gave a slightly poorer fit, but both human models with the interacting heads motif give very poor R-factors compared to the structure from Hudson et al. [10]. The R-factor for the chimera model was slightly improved if the 2D temperature factor was included (see text).

For comparison, the diffraction pattern of the thick filament model from Hudson et al. [10] (Figure 3a) was computed in exactly the same way using the same programs in the same unit cell, with R-factors, as above, being computed in each case (Figure 4). Note that the best model of AL-Khayat and Squire [31] gave similar results.

### 2.5. Vertebrate Muscle Myosin Filaments

**Figure 5:**
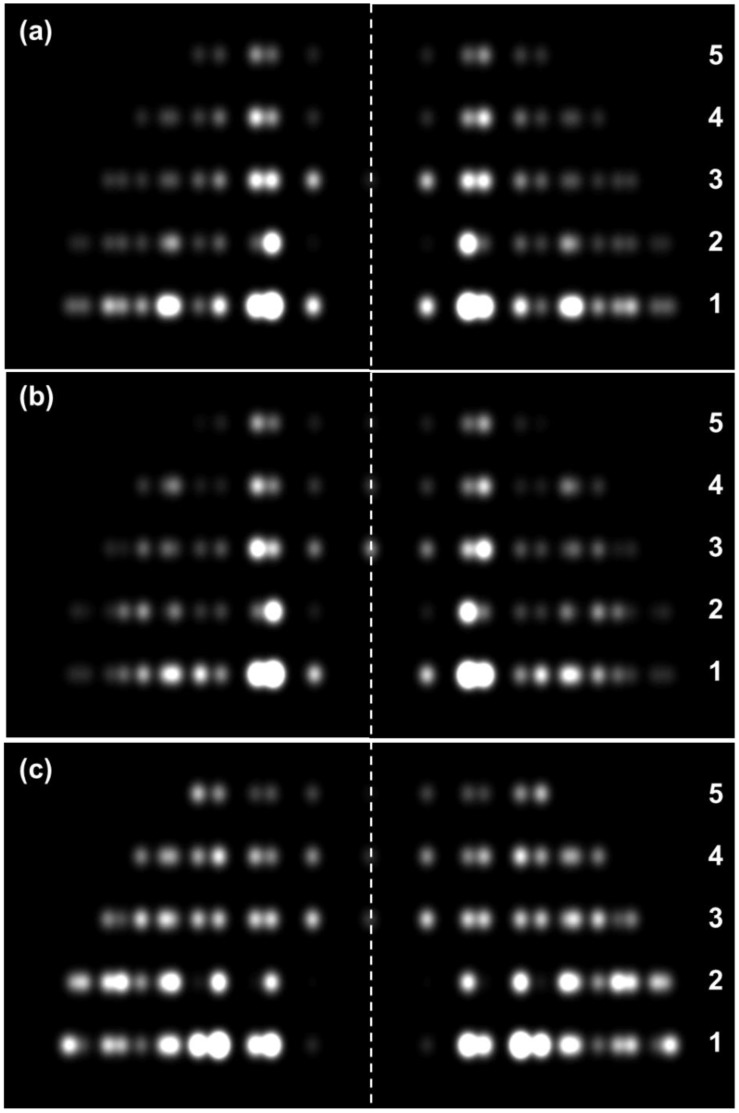
(**a**) Simulated X-ray diffraction pattern using the observed corrected intensities from bony fish muscle (cf. Figure 2a). (**b**) Calculated diffraction pattern using the myosin filament structure (Figure 3a) from Hudson et al. [10] that is the best fit to (**a**); R-Factor 7.9%. (**c**) The calculated X-ray pattern if the human heart muscle myosin filaments with the interacting head motif (AL-Khayat et al. [26]) are put into the fish muscle unit cell instead of the structure from Hudson et al.; lowest R-Factor = 54%. Inclusion of myosin S2 in the calculation in (**c**) makes the fit slightly less good (see Figure 4). All patterns were scaled to have the same total intensity. Introduction of a small lateral disorder factor (2D temperature factor) slightly improved the R-factor in (c) (with S-2) to 32% with a root mean squared lateral displacement of the myosin filament from its ideal lattice point of 22 Å.

Figure 5 compares the observed X-ray diffraction pattern from resting bony fish muscle (a) with the computed diffraction patterns from the model of Hudson et al. (b) [10] (Figure 3b,c), a hybrid structure with the EM density map of AL-Khayat et al. [26] placed in the fish muscle unit cell (Figure 3a). The best (lowest) R-factors obtained were R = 0.0786 (or 7.9%) for the model of Hudson et al. [10] and R = 0.54 (54%) for the human thick filament reconstruction in the fish muscle unit cell. Note that the R-factor value for fish muscle is different here from in Hudson et al. ([10]; 3%) because we are using a slightly different R-factor definition (see Equation (3)). Clearly the human structure is a relatively poor fit to the observed pattern, as Figure 4 and Figure 5 show. Figure 4 shows the variation of the R-factor with the rotation of the human thick filament models in the fish muscle lattice. This is a periodic function with equivalence every 60° because of the symmetry of the system (i.e., a hexagonal lattice, and a motif with approximate 9-fold rotational symmetry in projection down the c-axis). For the human chimera structure, the R-factor varied from R = 54% at its best to R = 78% at its worst. Even the best value is poor compared with the structure from Hudson et al. [10]. The fit was slightly worse (R = 56%) when the S2 part of myosin was included in the heart muscle structure as well (Figure 4). It is not clear whether S-2 should be included as part of the regular backbone or as part of the more variable head organisation on the three crowns. The fits were slightly better if a 2D lateral disorder factor (temperature factor) was included in (c) to give R = 32% rather than 56% with S-2 included. The mean radial displacement was 22 Å.

Note that AL-Khayat and Squire [31] carried out a search around the IHM structure in their earlier analysis of the fish muscle diffraction pattern and found, as here, that they were unable to get a good fit to the observed diffraction data.

Figure 3b shows the appearance of the human thick filament structure [26] in the fish muscle unit cell with the preferred rotation of the thick filament in the lattice. Also shown are the actin filaments. For comparison Figure 3a shows the myosin filament structure from Hudson et al. [10] in the same unit cell. In Figure 3a the actin binding domains on some of the myosin heads are close to their actin binding sites, but these sites are shielded from the actin filaments in the IHM structure in Figure 3b.

### 2.6. The Insect Flight Muscle Unit Cell

**Figure 6:**
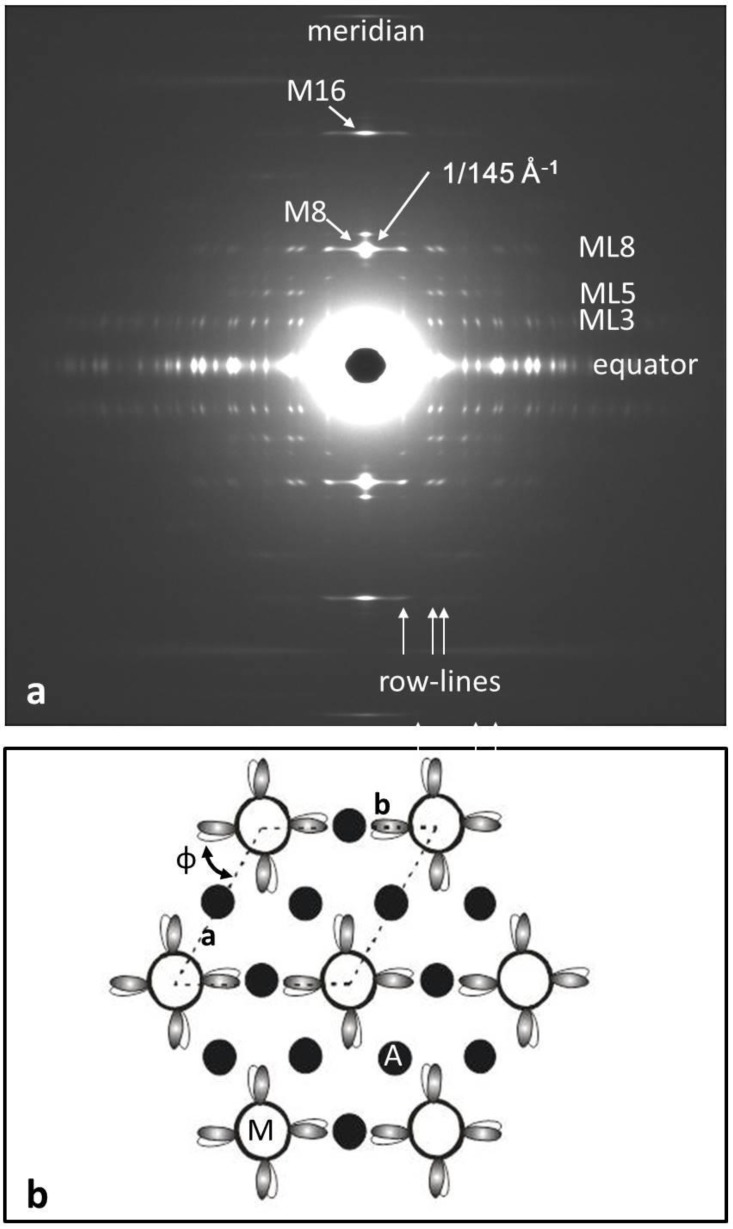
(**a**) The observed low-angle X-ray diffraction pattern from insect flight muscle (*Lethocerus*; fibre axis vertical) courtesy of R.J. Edwards and M.K. Reedy [7,8,9] and (**b**) the insect flight muscle unit cell viewed down the fibre axis, showing the unique orientation of the myosin filaments in the lattice and their 4-fold rotational symmetry on a single crown of heads. Myosin heads are represented as shaded or white ovals. Actin filaments, all halfway between adjacent myosin filaments, are labelled as A. Compare Figure 2b for vertebrate striated muscles. The rotation **N** in the lattice is ill-defined (see text).

X-ray diffraction patterns from resting insect flight muscle (Figure 6a) were kindly provided by R.J. Edwards (Duke University, Durham, NC, USA; [7,8,9]). These were stripped by us using both the FibreFix program [28] and using Fiji [32] followed by PeakFit (https://systatsoftware.com/products/peakfit/). The measured unit cell dimensions (Figure 6b) were: a = b = 510 Å, c = 1160 Å, α = β = 90°, γ = 120°. There is one myosin filament per unit cell, but there are three actin filaments to every myosin and these are halfway between neighbouring myosin filaments (not at the trigonal points found in vertebrate muscle: see Figure 2b). As in the case of our analysis of resting vertebrate muscle, diffraction patterns were computed by placing various versions of the resting insect myosin filaments into the insect flight muscle unit cell and computing the structure factor F(*h*,*k*,*l*) as in Equation (1). Once again, in principle, the rotation of the filament in the lattice is important, but the insect thick filament has 32-fold rotational symmetry in projection down the c-axis, so in a six-fold symmetric lattice a rotation range of only around 360/192~2° is needed to calculate the expected intensities. At the resolution that we are considering, about 60 Å, such a rotation has very little effect.

### 2.7. Insect Flight Muscle Myosin Filaments

**Figure 7:**
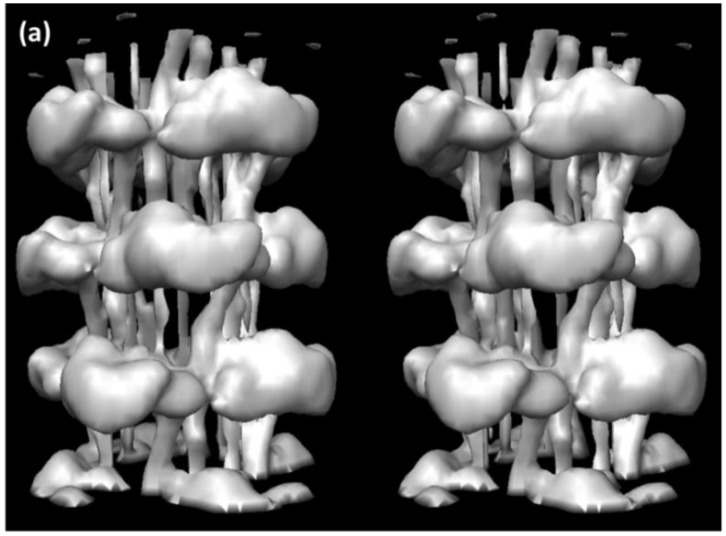
Part of the 3D reconstruction of the insect flight muscle myosin filament according to Hu et al. [27], here showing slightly more than three crowns as a stereo (wall-eyed) surface view.

A 3D reconstruction of the thick filaments from *Lethocerus* flight muscle (Figure 7) was published by Hu et al. in 2016 [27]. This reconstruction had the remarkable property that the backbone structure was resolved to 6-Å resolution, enough to reveal the curved molecular crystal packing of the α–helical coiled-coil myosin rods in the backbone [30,33]. However, the head pairs were only resolved to about 25-Å resolution, presumably because of some inherent disorder in the head organisation. Because of a technical problem, they were unable to supply us with the coordinates of the head pairs. We have, therefore, used the protein density map produced by Hu et al. [27] (kindly provided to us by Kenneth Taylor, Florida State University) as a starting point with which to investigate the head arrangement in resting insect flight muscle.

**Figure 8:**
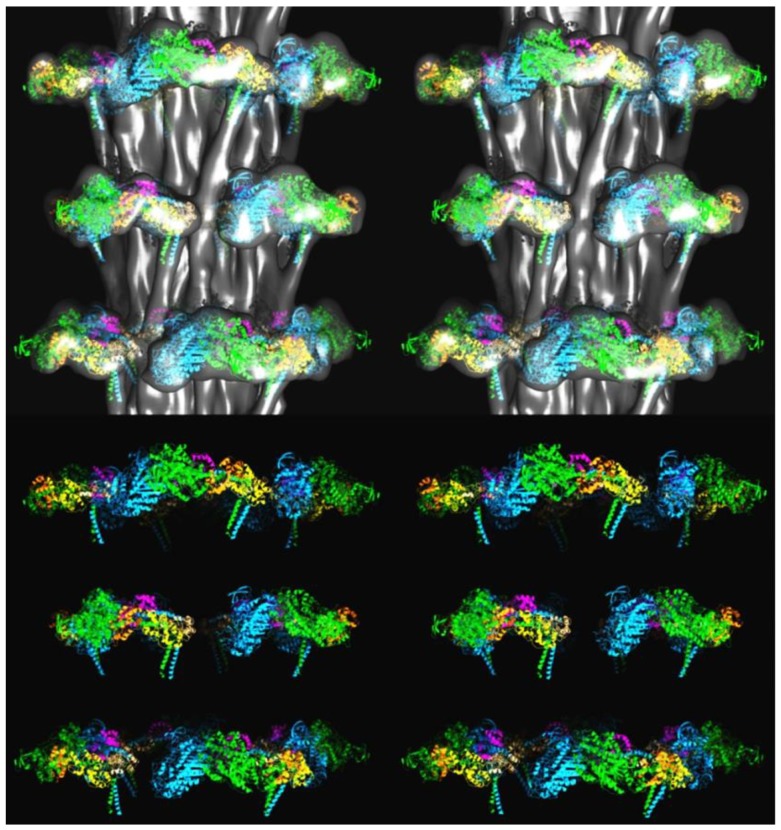
3D structure of the insect flight muscle myosin filament according to Hu et al. [27] shown as stereo (wall-eyed) side views containing three crowns. (**Top**) Surface view of the density map, low pass Fourier filtered to 25-Å resolution, with fitted coordinates of atomic models of myosin heads in the interacting head motif arrangement, as determined in the present work, shown in cartoon representation. (**Bottom**) The atomic models without the density map. Coordinates colour-coded as follows: Free head heavy chain—cyan, essential light chain—magenta, regulatory light chain—straw: Blocked head heavy chain—green, essential light chain—orange, regulatory light—yellow.

**Figure 9:**
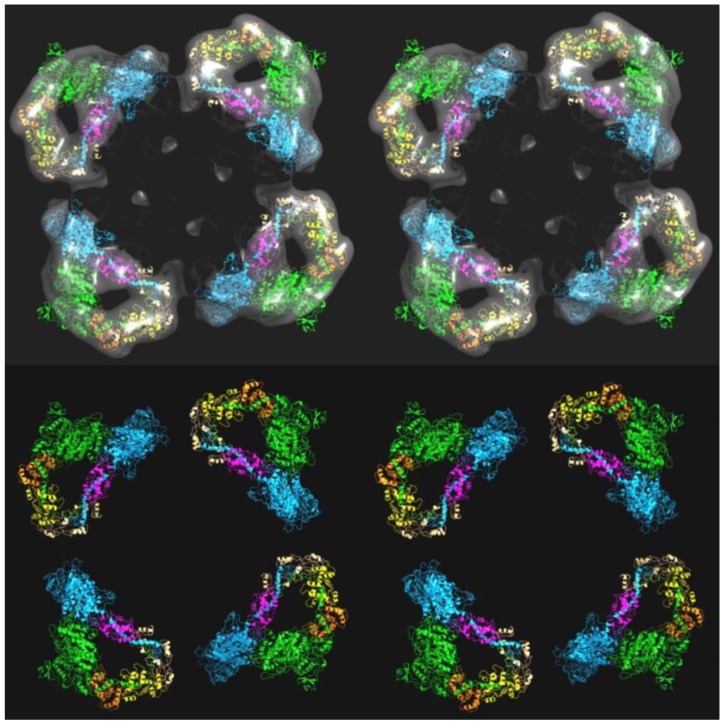
The models in Figure 8 shown looking down the filament axis, with the same colour coding. (**Top**) Density plus fitted model; (**Bottom**) Fitted model only. The head pairs form a good fit with a closed structure, as in the interacting head motif.

Hu et al. [27] suggested that, like thick filaments in other resting muscles, the observed densities could be described by the interacting head motif arrangement originally described by Wendt et al. [21]. As noted above, models based on the interacting head motif arrangement have been found to match well with electron microscope-derived 3D reconstructions of, for example, tarantula [22], scorpion [34], and vertebrate cardiac thick filaments [24,25], including human thick filaments [26]. Accordingly, we developed a model for the head arrangement in the insect flight muscle thick filament density map using as a starting point the atomic coordinates fitted to the tarantula thick filament ([22], pdb accession code 3DTP).The tarantula interacting head motifs were initially fitted as rigid bodies within the crown domains using Chimera [35]. Subsequent conformational optimization was achieved with COOT [36] and flexible fitting with MDFF [37] giving rise to the structure illustrated in Figure 8 and Figure 9. The coordinates of the fitted model are well contained within and account well for the protein density of the crown domains. As in the interacting head model of Hu et al. [27], the long axes of both heads are oriented approximately perpendicular to the filament axis. The free head is adjacent to and runs at a tangent to the thick filament backbone (Figure 9). The blocked head is located at higher radius: its lever arm is angled outwards diverging to higher radius while the motor domain is curved back in to interact with the motor domain of the free head. **PDB Deposition:** The coordinates of the insect flight muscle myosin filament head arrangement defined in this paper have been deposited at the Protein Data Bank as PDB ID 6SO3.

As described in detail by AL-Khayat et al. [11], the insect flight muscle structure gives rise to layer-lines that are orders of the 1160-Å axial repeat. From the symmetry of the myosin filament, the strong layer-lines are at (*l* =) 3 (387 Å), 5, 8 (145 Å: meridional, M8, and layer-line), 11, 13, 16 (72.5 Å; meridional, M16, and layer-line) and so on. The complication is that the actin filaments in insect flight muscle also have a pseudo-repeat of around 387 Å, so they also contribute to the third layer-line of the 1160-Å repeat, as does troponin which lies on a 385-Å repeat. This means that the third layer-line is not useful for defining the myosin filament structure unless the whole unit cell is modelled including actin, tropomyosin, and troponin and this adds many more parameters to be fitted. For this reason we chose to assess possible insect myosin filament structures using the layer-lines listed above apart from layer-line *l* = 3. As in the case of the vertebrate muscle, there are multiplets at the same radius (R) and the intensities being fitted are the sums of all the peaks in the same multiplet.

### 2.8. Is the Interacting Myosin Head Motif Present in the Unit Cell of Relaxed Insect Flight Muscle

**Figure 10:**
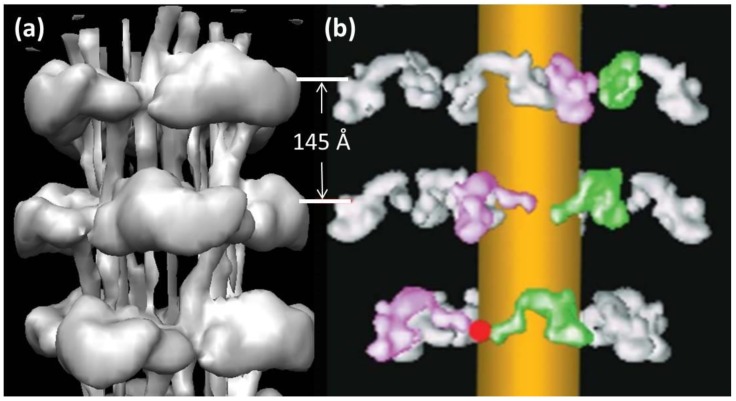
Comparison of three crown levels of the Hu et al. [27] insect flight muscle myosin filament density map (**a**) and the model (**b**) from AL-Khayat et al. [11] based on fitting X-ray diffraction data.

**Figure 11:**
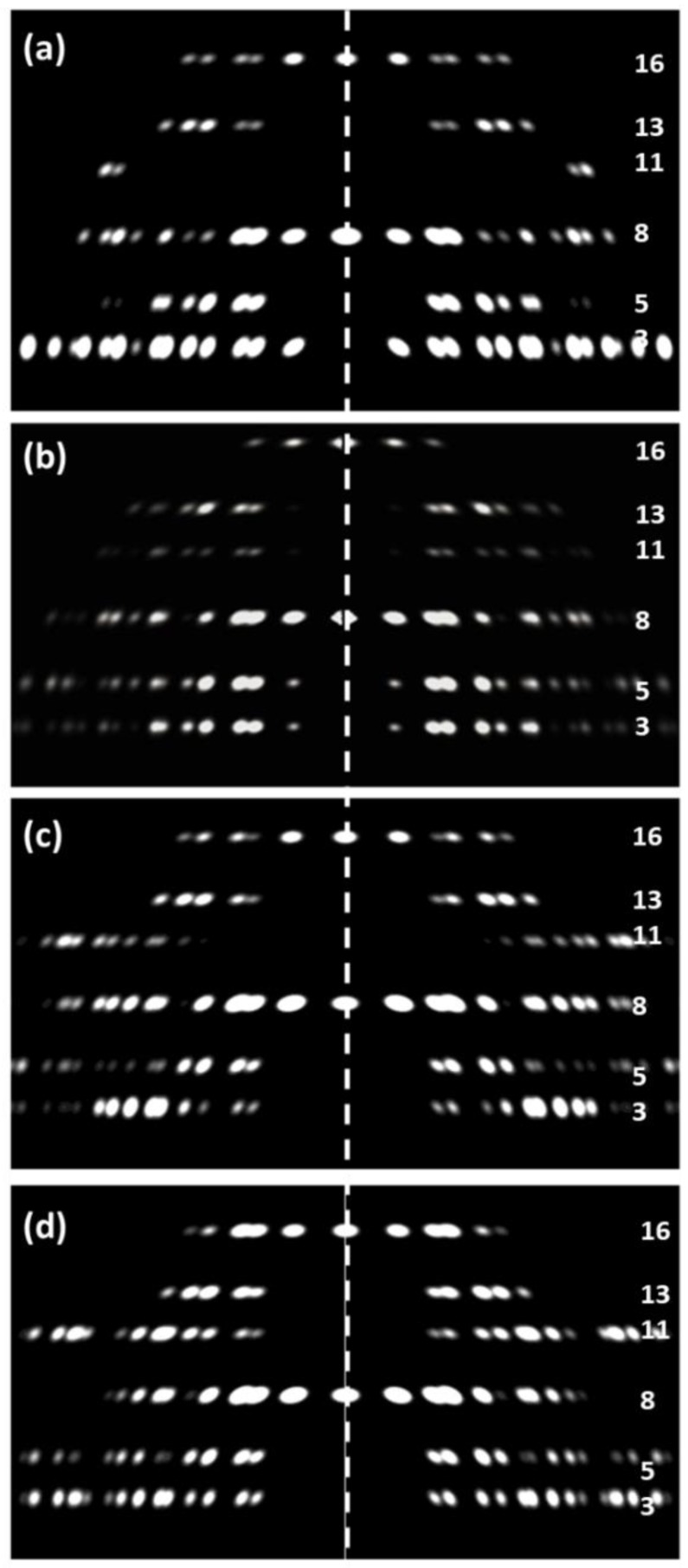
Comparison of the observed low-angle X-ray diffraction pattern (**a**) from relaxed insect (*Lethocerus*) flight muscle after processing (upper half of Figure 6a) and the simulated diffraction patterns from various versions of the insect myosin filament in the unit cell: (**b**) The published model from AL-Khayat et al. [11], R-factor = 9.7%; (**c**) The density map from Hu et al. [27] (Figure 7) put into the insect unit cell; R-factor = 33%, (**d**) The fitted atomic model with the interacting heads motif (Figure 8 and Figure 9), R-factor = 27%. (**b**–**d**) used the R-factor given in Equation (3). Layer-line numbering is based on the approximate 1160-Å axial repeat in insect flight muscle. Layer-line 3 was not used for the R-factor calculations, because both actin and troponin contribute to that layer-line as well. All patterns were scaled to give the same total intensity in the pattern, apart from (**b**) which is modified from the results of AL-Khayat et al. [11] and scaled to look similar to the other patterns. Introduction of a lateral (2D) disorder factor slightly improved the R-factors in (c) and (d) to 21% and 19% with a root mean squared displacement of the myosin filament from its ideal lattice point of 18 Å and 17 Å respectively.

The resting insect flight muscle myosin filament structure has previously been modelled by AL-Khayat et al. [11] to give the structure shown in Figure 10b. In order to test whether the structure from Hu et al. [27] can better explain the observed low-angle X-ray diffraction pattern from insect (*Lethocerus*) flight muscle (Figure 6a), we have put the density map from Hu et al. [27] into the insect unit cell and calculated its expected X-ray diffraction pattern. We have also done the same with our new atomic model (Figure 8 and Figure 9) of the interacting heads motif structure fitted to the density from Hu et al. The results are shown in Figure 11 in comparison with the model from AL-Khayat et al. [11].

Note that the original AL-Khayat et al. modelling used a slightly different R-Factor from the one quoted as Equation (2) above. This is shown as Equation (3). It includes the standard deviations (Φ) of the observed intensities.


R = Σ[(I_obs_ − I_calc_)^2^/Φ^2^]/Σ(I_obs_)^2^/Φ^2^] (3)

Usually the most intense peaks are better determined than the weak ones, so the effect of including the Φ^2^ factors is often not large. We have used Equation (3) throughout our analysis of the insect flight muscle diffraction patterns. The result is that the R-factors were 9.7% for the published structure from AL-Khayat et al. [11], 33% for the density map from Hu et al. [27], and 27% from the PDB fit to the density map from Hu et al. in the present work as in Figure 8 and Figure 9. Even with the introduction of a 2D disorder function (2D temperature factor), the R-factors with the insect density map and IHM model only reduce to 21% and 19% respectively. The interacting head motif structure is a relatively poor fit to the observations from resting insect flight muscle.

## 3. Discussion

**Figure 12:**
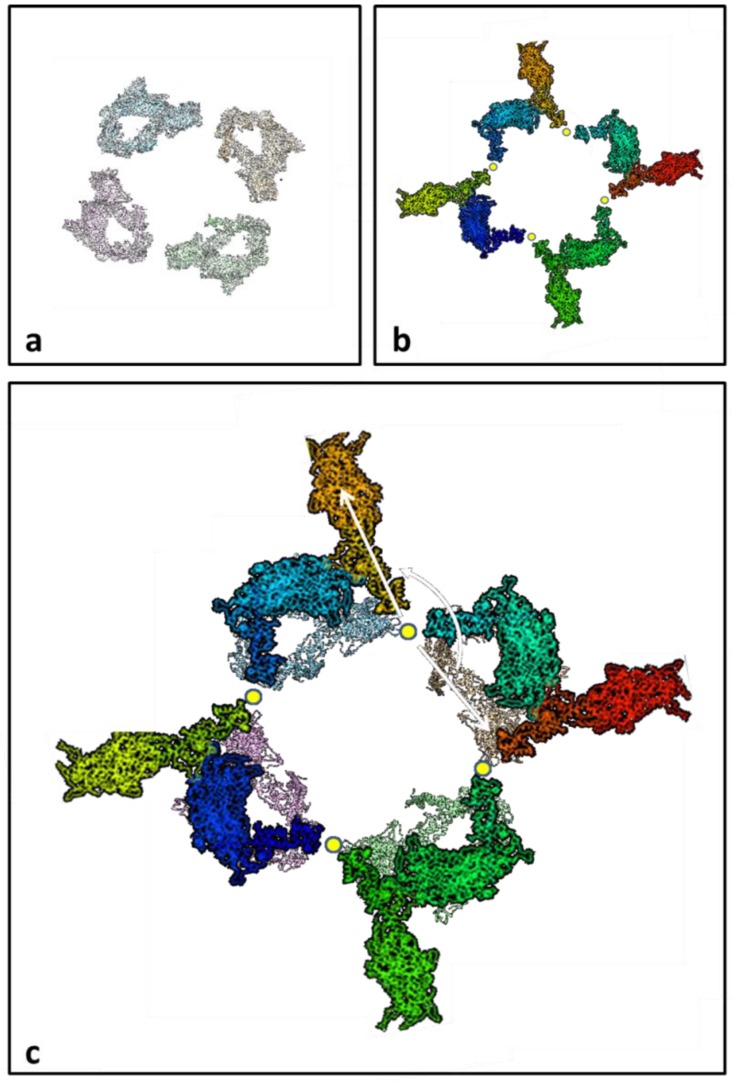
(**a**) One crown of the IHM (super-relaxed?) head configuration on the myosin filaments of insect flight muscle, looking M-wards and showing the interacting head motif (cf. Figure 9). (**b**) One crown of the resting insect flight muscle myosin filament (slightly refined from) reported by AL-Khayat al [11], at the rotation around its long axis that best fits with the structure from Hu et al. [27], with the S2 positions coincident and showing one head near the filament surface and the other projecting radially outwards from the filament backbone (shown here as an empty space). (**c**) The structures in (**a**,**b**) are superimposed to show the similar, overlapping, locations of one of each head pair and the changed positions of the other heads between the two structures. The white arrows indicate, for the free head swing model, the swing of the free heads around the S2 positions, which are shown as small yellow dots, between what we assume is the super-relaxed state and what we now think of as the “activated” relaxed state. In an alternative mechanism (not illustrated), the head switch mechanism, the outer blocked head in the IHM structure moves to project radially outwards and the inner free IHM head moves out to the position originally occupied by the blocked head.

We have shown in our analysis that the interacting head motif structures for the myosin filaments in a vertebrate muscle (bony fish) [24,25,26] and an insect flight muscle (*Lethocerus*) [27] do not explain the X-ray diffraction observations previously recorded from these same muscles in the resting state [6,11] as well as our original fitted structures [10,11]. We do not know if this is a general result for all vertebrate striated muscles and insect flight muscles, but analysis of other muscles is beyond the scope of the present paper. However, since it occurs in two such radically different muscle types, the ‘heads out’ structures (Figure 3a and Figure 12b) may be a consistent feature of intact relaxed muscles. It should be remembered, though, that for the vertebrates we are comparing a fish muscle diffraction pattern and human cardiac muscle thick filaments. They could have different structures.

It would appear that, under some circumstances, the super-relaxed state, which we take to be the interacting head motif structure, may occur and that this reduces the resting acto-myosin ATPase to conserve energy. On the other hand, dissected fish and insect muscles kept in a normal relaxed state have different structures, which we will call the *activated relaxed state*. Something along these lines has previously been reported by Ma et al. [38] using mouse muscle. They also treated their muscles with blebbistatin and reported that this shifted the relaxed state towards the super-relaxed state. Blebbistatin is known to inhibit the myosin ATPase [39,40].

Why should X-ray diffraction and electron microscopy show different results? The most obvious difference in the myosin filament situation is that the X-ray diffraction data are from intact muscle where the myosin filaments are in a lattice close to actin filaments and that the activated relaxed structure may be stabilized by the presence of the lattice. This may not be a structure that can be readily preserved in electron microscopy preparations and is therefore never seen. The interacting head motif structure that has been seen ubiquitously in recent electron microscopy studies may be such a well-organised, compact structure that it lends itself to relatively easy preservation for electron microscopy. Further work is needed to see if intact muscles can be sufficiently pushed into the super-relaxed state so that they give the X-ray diffraction patterns shown as Figure 5c for vertebrate or Figure 11c,d for insect flight muscle.

We show elsewhere [41] that when bony fish muscle is fully active there is still a sampled myosin layer-line pattern that is not just a reduced version of the resting pattern. If there had been some fibres that for some reason had not been activated, a remnant myosin layer line pattern might be expected. However, the analysis of Eakins et al. [41] shows that the myosin layer-lines in the active pattern are quite different from the resting layer-lines. The active layer-lines show evidence of a new “active” myosin-centred structure. So, in bony fish muscle, there may be an ordered super-relaxed state under some conditions (one that we have not yet seen), an “activated” relaxed state (the present work and Ma et al. [38]), and an ordered, myosin-centred, active state (Eakins et al. [41]), possibly with weak-binding and pre-powerstroke heads. In addition, there are strong head states on actin that are involved in force production. Ma et al. [38] also found myosin layer-lines in their active patterns from mice, so their results and ours suggest that these three myosin-centred states may be a general feature of vertebrate muscles. It also means that, apart from the super-relaxed state, the other resting myosin filament states are not just disordered states as has been thought before, but are specifically-ordered “activated” states.

Turning now to insect flight muscle, the same story appears to hold true there. The compact interacting head motif structure is not the best fit to the observed X-ray diffraction data. The preferred structure that better fits the layer-line pattern [11] has one of the bridges extending radially outwards towards actin (Figure 12b), with the other head closer to the backbone in the same position as the outer head in the IHM structure in Figure 12a. 

If the super-relaxed state from Hu et al. [27] occurs in intact insect flight muscle, how would the heads move to generate the structure seen by AL-Khayat et al. [11]? Figure 12 shows what might happen. It could be that the inner free head in Figure 12a rotates around the S2 by almost 180° to get to the activated state (Figure 12b; Figure 12c arrow). The blocked head, on the other hand, would then stay in almost the same place in both the super-relaxed state and the “activated” relaxed state (Figure 12a,b). The head movements required to convert from one relaxed structure to the other in this scenario, which we call the free head swing model, are indicated in Figure 12c, where the IHM and AL-Khayat et al. [11] head arrangements are superimposed. The alternative to this is that the blocked head in Figure 12a moves to project outwards as in Figure 12b while the free head in (a) moves to the position originally occupied by the blocked head. We call this the head switch mechanism. In either case, activation puts one of each head pair very close to an actin filament. The radially projecting head in Figure 12b may well still be stabilised by an interaction with the other head, but one that is different from the IHM structure.

## 4. Conclusions

Using further analysis of the low-angle X-ray diffraction data from bony fish muscle and insect flight muscle it has been demonstrated that the normal resting state of in both intact muscles in the published X-ray diffraction experiments is probably not the IHM, super-relaxed state that has been seen in 3D single particle reconstructions from electron microscopy. There must be two different structures, both “relaxed” and both ordered; the super-relaxed IHM state and the previously modelled structures of Hudson et al. [10] and AL-Khayat et al. [11]. The drug blebbistatin can encourage the filaments to favour the super-relaxed configuration [39,40], but what causes this transition in the muscle? Particularly noteworthy is the fact that the previous X-ray diffraction modelling of the head positions in relaxed fish muscle by Hudson et al. [10] and in relaxed insect flight muscle by AL-Khayat et al. [11] appears to be satisfactory. Both give relatively low R-factors. This means that the powerful X-ray diffraction modelling that these two studies involved can be applied to time-resolved X-ray diffraction data from active muscle to give “Muscle—the Movie” [2,42], as further discussed in Eakins et al. [41]. Until electron microscopy can be applied in a fast time-resolved manner, and this prospect still seems a long way off, this may be the only way to actually visualise the molecular changes in the acto-myosin system as force is generated.

## Abbreviations

IHM	Interacting heads motif
IFM	Insect flight muscle
EM	Electron microscopy
ATP	Adenosine triphosphate
ADP	Adenosine diphosphate
Pi	Inorganic phosphate.
